# Sleep Problems and Sleep Quality in the General Adult Population Living in South Tyrol (Italy): A Cross-Sectional Survey Study

**DOI:** 10.3390/clockssleep7020023

**Published:** 2025-05-08

**Authors:** Dietmar Ausserhofer, Giuliano Piccoliori, Adolf Engl, Pasqualina Marino, Verena Barbieri, Stefano Lombardo, Timon Gärtner, Christian J. Wiedermann

**Affiliations:** 1 Institute of General Practice and Public Health, Claudiana, 39100 Bolzano-Bozen, Italy; giuliano.piccoliori@am-mg.claudiana.bz.it (G.P.); adolf.engl@gmail.com (A.E.); pasqualina.marino@am-mg.claudiana.bz.it (P.M.); verena.barbieri@am-mg.claudiana.bz.it (V.B.); christian.wiedermann@am-mg.claudiana.bz.it (C.J.W.); 2 Claudiana Research, College of Healthcare Professions, 39100 Bolzano-Bozen, Italy; 3 Provincial Institute of Statistics (ASTAT), 39100 Bolzano-Bozen, Italy; stefano.lombardo@provincia.bz.it (S.L.); timon.gaertner@provinz.bz.it (T.G.)

**Keywords:** adults, sleep quality, sleep behaviors, cross-sectional study, population-based survey, stratified probabilistic sampling

## Abstract

It remains unclear how sleep health has developed in the general population after the COVID-19 pandemic. This study aimed to (1) investigate the prevalence of sleep problems and poor sleep quality and (2) explore the associated sociodemographic and health-related factors in South Tyrol, Italy. A cross-sectional, population-based survey was conducted with a stratified probabilistic sample of 4000 adults aged ≥ 18 years. Sleep quality was assessed using the brief version of the Pittsburgh Sleep Quality Index. Descriptive and logistic regression analyses were performed to analyze the data. A total of 2090 adults (53%) completed the survey. Poor sleep quality was reported by 17.8%, with 28.2% of participants reporting insufficient sleep duration (i.e., six hours or less), 12.7% having problems staying asleep (i.e., waking up to 3–4 times a week and unable to fall asleep again), and 8.7% having problems falling asleep (i.e., >30 min). Sleep problems and poor sleep quality were associated with sociodemographic and health-related factors, including gender, age, mother tongue, chronic disease, and sleep hygiene. Notably, Italian-speaking participants reported poorer sleep quality and greater difficulty staying asleep compared to German-speaking participants, highlighting potential sociocultural influences on sleep health. This study contributes to understanding the unique sleep health challenges in a multilingual region, highlighting the role of sociocultural factors in sleep quality differences between language groups.

## 1. Introduction

Sleep is a fundamental biological process essential for human health and well-being [1,2]. Sleep health, which involves adequate sleep quality and quantity, is crucial for maintaining physical and mental health, cognitive function, and overall quality of life [3,4]. Currently, there is a vast amount of evidence indicating that sleep disorders and poor sleep quality are associated with physical health outcomes, including poor cardiovascular health, high blood pressure, obesity, and diabetes [5,6,7,8]; mental health consequences such as depression, anxiety, and suicidal behavior [9,10,11]; and overall mortality [12,13]. Moreover, the negative impact of sleep problems extends beyond individual health, affecting workplace productivity [14], healthcare costs [15], and public safety [16].

According to the American Academy of Sleep Medicine and Sleep Research Society, 7–8 h of total sleep is recommended as an adequate sleep quantity for the adult population [17]. Sleep quality is defined as an individual’s self-satisfaction with all aspects of their sleep experience [18]. The US National Sleep Foundation has put forward the four key indicators for good sleep quality: (i) less difficulty in initiating sleep, (ii) sleeping more time while in bed, (iii) less than two instances of night waking, and (iv) ability to go back to sleep within 20 min of a night awakening [19].

Sleep problems and poor sleep quality have become increasingly prevalent in modern society and affect a significant proportion of the global population [1]. Between 10% and 30% of the general adult population is affected by sleep problems and poor sleep quality [20,21], with a prevalence of diagnosed sleep disorders reported in the literature ranging between 5% and 15% [22,23,24]. The COVID-19 pandemic has had a significant impact on sleep quality and disturbances worldwide. A meta-analysis of 63 studies revealed a 40% higher risk of poor sleep quality during the pandemic than pre-pandemic [25], with increased stress and anxiety related to health concerns, economic uncertainty, social isolation, working from home, reduced physical activity, increased screen time, and COVID-19 infection itself as influential factors. Sociodemographic (e.g., women [26,27], older adults [28], economic status, and race/ethnicity [29,30]), health-related (chronic diseases and multimorbidity [31,32]), and environmental [30] factors are associated with sleep disorders and poor sleep quality.

While sleep research has been conducted in various populations worldwide [1], there is still limited information on sleep problems and sleep quality in Italy [33,34,35]. The most recent cross-sectional study, conducted in 2019, included a sample of 3120 subjects from the Italian general population and found that 14.2% of Italian adults were dissatisfied with sleep, and 29.5% reported insufficient sleep duration [35]. South Tyrol, an autonomous province in northern Italy, has a unique cultural and linguistic landscape, with influences from both Italian and German-speaking populations, with distinct cultural practices, healthcare beliefs, and social norms. However, no data on sleep problems or sleep quality are currently available.

Given the importance of sleep in health, it is vital to monitor the prevalence of sleep quality and sleep problems at the population level and compare these estimates and trends within and between countries [1]. However, it remains unclear how sleep quality and sleep problems have developed in the general population of Italy and other countries after the COVID-19 pandemic. South Tyrol experienced significant social stress during the COVID-19 pandemic, driven by mistrust in national health measures and low vaccination rates, particularly among the German-speaking population. The region’s unique sociopolitical context, including a history of political autonomy movements and reliance on Austrian healthcare practices, contributed to vaccine hesitancy. These factors were compounded by the spread of misinformation and conspiracy theories, which led to lower vaccine acceptance compared to other regions in Italy. As observed in recent surveys, these local dynamics played a crucial role in shaping public health responses during the pandemic, influencing both vaccination uptake and broader health behaviors [36].

Research in such regions can provide insights into the intersection of culture, language, and health outcomes, particularly in sleep research. The coexistence of two linguistic groups, approximately 70% of the population speaking German and 25% speaking Italian, within the same geographical region presents a valuable opportunity to investigate health-related behaviors, including sleep quality, in a population influenced by diverse cultural factors. More precisely, examining sleep patterns in this context can contribute to a more comprehensive understanding of sleep health in diverse populations and inform public health initiatives to improve sleep health tailored to multilingual and multicultural populations.

Therefore, this study aimed to (1) describe the prevalence of poor sleep quality and sleep problems among adults living in the culturally distinct Autonomous Province of Bolzano (South Tyrol, Italy) and (2) explore the sociodemographic and health-related factors associated with sleep quality, with a specific focus on the potential influence of linguistic and cultural differences between German- and Italian-speaking groups.

## 2. Results

### 2.1. Sample Characteristics

Of the 4000 individuals invited, approximately 2120 returned the questionnaire, yielding a response rate of 53%. Among these, 2090 adults provided fully completed responses and were included in the analysis, corresponding to a completion rate of approximately 98.6%.

As described in Table 1, most participants were female (55.2%), 55 years of age or above (50.7%), German-speaking (66.9%), not living alone (81.7%), and had the highest educational level below high school (55.7%). This level refers to participants who completed middle school or vocational school but did not attain a high school diploma. In South Tyrol, vocational school programs, which typically start after completing middle school (at around age 14 or 15), provide specialized training in technical or professional fields. These individuals may have gained qualifications through vocational training without completing a high school diploma.

The urban sample included 381 participants, while the rural sample included 1709 participants. The gender distribution was similar in both groups, with 61.7% of the urban sample and 53.7% of the rural sample being female. In terms of age, the urban group had a higher proportion of older adults: overall, 58.8% of the urban sample were aged 55–99 years, compared to 48.9% in the rural sample. The rural group had a higher proportion of younger adults, with 18.9% of participants aged 18–34 years, compared to 15.7% in the urban sample. Regarding language, 24.1% of urban participants identified as German-speaking, while 65.9% were Italian-speaking. The rural group was predominantly German-speaking, with 76.4% of rural participants identifying as German-speaking and 14.5% as Italian-speaking.

Overall, 61.9% reported good or very good health, and 61.3% had no diagnosed chronic disease. Among the participating adults, 80.8% often or always cared for enough sleep, and 82.9% never took sleep medications (as natural supplements or by prescription) over the last month (see Table 1).

### 2.2. Sleep Quality and Sleep Problems

Among the 2090 participants, 17.9% (95% CI: 16.2–19.4) reported their overall sleep quality as either “quite bad” or “very bad.” The median sleep duration was 7.0 h per night (IQR: 2.0 h), with 28.2% (95% CI: 26.3–30.1) of the participants reporting insufficient sleep duration, defined as 6 h or less per night. Regarding sleep disturbances, 12.7% (95% CI: 11.3–14.1) reported experiencing problems staying asleep, defined as waking up 3–4 times per week and being unable to fall back asleep. Additionally, 8.7% (95% CI: 7.5–9.9) of the participants reported difficulties falling asleep, taking more than 30 min to do so.

### 2.3. Sociodemographic and Health-Related Factors Associated with Poor Sleep Quality and Sleep Problems

Table 2 (a,b) present the bivariate differences in sleep duration, sleep quality, problems falling asleep, and staying asleep across demographic groups. A higher proportion of females showed poor sleep quality, problems falling asleep, and staying asleep compared to males. Age differences were observed, with the 55–99-year-old group reporting the shortest sleep duration, poorer sleep quality, and more often having problems staying asleep. However, younger adults (18–34 years) had the highest proportion of those having problems falling asleep. Individuals with an educational level below high school reported more often having poor sleep quality and problems staying asleep compared to those with an educational level of high school or higher.

Italian-speaking participants reported shorter sleep durations than German-speaking ones and showed a higher prevalence of poor sleep quality, as well as problems staying asleep. The “other language” group showed sleep characteristics similar to German speakers, with comparable sleep duration, problems staying asleep, and proportion of individuals with poor sleep quality; however, they had more problems falling asleep. Rural residents reported longer sleep durations than urban residents and a higher proportion of individuals with poor sleep quality, but no differences were observed in problems falling asleep and staying asleep.

Multivariable logistic regression analysis revealed several factors associated with poor sleep quality and sleep problems, such as insufficient sleep duration, problems getting to sleep, and problems staying asleep Table 3 (a,b).

Compared to males, females had nearly two times higher probability of reporting poor sleep quality (OR: 2.03, 95% CI: 1.63–2.55, *p* < 0.001), problem staying asleep (OR: 1.96, 95% CI: 1.48–2.61, *p* < 0.001), and problems falling asleep (OR: 1.91, 95% CI: 1.37–2.69, *p* < 0.001) but not insufficient sleep duration (OR: 1.07, 95% CI: 0.86–1.33, *p* = 0.6). Older adults (55 years and above) were more likely to report poor sleep quality (OR: 1.69, 95% CI: 1.20–2.39, *p* = 0.003), insufficient sleep duration (OR: 1.90, 95% CI: 1.35–2.70, *p* < 0.001), and problems staying asleep (OR: 1.72, 95% CI: 1.09–2.79, *p* < 0.023) compared to the youngest age group (18–34 years). Both adults aged 35–54 years (OR: 0.42, 95% CI: 0.26–0.57, *p* < 0.001) and adults aged 55 years or older (OR: 0.60, 95% CI: 0.38–0.94, *p* = 0.024) had lower odds of falling asleep than those aged between 18 and 34 years. Italian-speaking adults were more likely to report poor sleep quality (OR: 1.40, 95% CI: 1.05–1.86, *p* = 0.023) and insufficient sleep duration (OR, 1.62; 95% CI: 1.22–2.16, *p* < 0.001) compared to German speakers, but this was not significant for problems getting to sleep and problems staying asleep. Higher education (high school or higher) reduced the odds of poor sleep quality (OR: 0.77, 95% CI: 0.60–0.97, *p* = 0.027) and problems staying asleep (OR: 0.60, 95% CI: 0.44–0.81, *p* < 0.001) but not for insufficient sleep duration (OR: 1.08, 95% CI: 0.85–1.37, *p* = 0.5) and problems falling asleep (OR: 0.77, 95% CI: 0.54–1.09, *p* = 0.15).

Adults reporting good or very good health were less likely to report poor sleep quality (OR: 0.51, 95% CI: 0.41–0.65, *p* < 0.001), problems staying asleep (OR: 0.54, 95% CI: 0.41–0.72, *p* < 0.001), problems getting to sleep (OR: 0.50, 95% CI: 0.35–0.70, *p* < 0.001), and insufficient sleep duration (OR: 0.76, 95% CI: 0.60–0.96, *p* = 0.024) compared to adults with poor or moderate health status. Adults with a diagnosed chronic disease had higher odds of poor sleep quality (OR: 1.31, 95% CI: 1.03–1.67, *p* = 0.027) and problems staying asleep (OR: 1.58, 95% CI: 1.18–2.14, *p* = 0.003) but not problems falling asleep (OR: 2.03, 95% CI: 1.41–2.90, *p* < 0.001) and sleep duration (OR: 1.37, 95% CI: 1.03–1.81, *p* = 0.028).

Often or always taking care to get enough sleep was significantly associated with lower odds of poor sleep quality (OR: 0.18, 95% CI: 0.14–0.23, *p* < 0.001), insufficient sleep duration (OR: 0.26, 95% CI: 0.20–0.35, *p* < 0.001), problems getting to sleep (OR: 0.33, 95% CI: 0.24–0.47, *p* < 0.001), and staying asleep (OR: 0.09, 95% CI: 0.07–0.12, *p* < 0.001). Using sleep medication once a week or more often over the last month was significantly associated with higher odds of poor sleep quality (OR: 2.27, 95% CI: 1.74–2.95, *p* < 0.001), insufficient sleep duration (OR: 1.37, 95% CI: 1.03–1.81, *p* = 0.028), problems getting to sleep (OR: 2.03, 95% CI: 1.41–2.90, *p* < 0.001), and problems staying asleep (OR: 1.67, 95% CI: 1.22–2.27, *p* < 0.001).

## 3. Discussion

In this cross-sectional study, we aimed to describe the prevalence of poor sleep quality and sleep problems among adults aged ≥ 18 years in South Tyrol, Italy, and to explore the associated sociodemographic and health-related factors. The findings highlight that 17.9% of the participants reported poor sleep quality, with insufficient sleep duration being the most prevalent sleep problem (28.2%). Problems related to sleep onset and sleep maintenance affected a notable proportion of the participants, with 12.7% reporting difficulty staying asleep and 8.7% experiencing trouble falling asleep. Sleep problems and poor sleep quality were associated with several sociodemographic characteristics, such as gender, age, native tongue, educational level, and health-related factors, including health status, chronic disease, taking care to get sufficient sleep, and intake of sleep medications.

One in six participants reported poor sleep quality, and over a quarter experienced insufficient sleep duration. Our results align with previous findings from Italy [35]. A 2019 study on a representative sample of 3120 adults reported that 14.2% of the Italian population experienced sleep dissatisfaction, and 29.5% had insufficient sleep (less than 6 h per night). Additionally, global estimates indicate that 13.3% of adults suffer from poor sleep quality, while 6.5% report insufficient sleep duration (less than 6 h per night) [20]. Similarly, a German study using the Pittsburgh Sleep Quality Index (PSQI) found that 36% of the general population experienced poor sleep quality [21]. These findings suggest that sleep problems, such as poor sleep quality and insufficient sleep duration, are common across different populations, with variation depending on sociodemographic factors such as age, gender, and health status.

Italy experienced a significant increase in insomnia prevalence during the COVID-19 pandemic, particularly affecting women, younger adults, and urban populations. Pre-pandemic insomnia rates (around 10% chronic and 30% with some symptoms) surged to higher levels (20–40% with significant insomnia, depending on the measure) during the lockdowns [37]. Post-pandemic, there has been some recovery toward normal sleep patterns; however, insomnia rates remain elevated compared to pre-pandemic levels [38,39]. The data from South Tyrol, with its higher proportions of poor sleep quality post-pandemic, could suggest that sleep health in this region might not have become fully “normalized” yet, as seen in the gradual recovery in other parts of Italy, which was studied by Salfi et al. [39]. Ongoing research and national health surveys are essential to determine whether insomnia prevalence will return to pre-pandemic levels or if a higher endemic level will persist in the population.

However, the substantial prevalence of poor sleep quality in South Tyrol warrants targeted interventions. There is a need to promote sleep health in public health agendas across the globe [1], including sleep health educational programs and awareness campaigns; increasing, standardizing, and centralizing data on sleep quantity and quality; and developing and implementing sleep health policies across sectors of society. Future research is needed to evaluate the outcomes and influences of such campaigns in improving sleep hygiene and literacy, as well as the diagnosis and treatment of sleep disorders, and reducing the onset and severity of comorbidities associated with disordered sleep [40].

Females disproportionately reported poor sleep quality, insufficient sleep duration, and problems with sleep initiation, consistent with previous research highlighting gender differences in sleep patterns [26,27]. While hormonal fluctuations, caregiving roles, and higher stress levels may explain these disparities, more research, from basic science to clinical research, is needed to gain a better understanding of the differences in sleep disorders between men and women in terms of prevention, clinical signs, treatment approaches, prognosis, and psychological and social impacts, to shape future therapeutic strategies [41]. Older adults (≥55 years) were more likely to report poor sleep quality, insufficient sleep, and problems staying asleep. As aging is associated with natural changes in sleep architecture [42], sleep problems and poor sleep quality are often under-recognized and undertreated in older adults, leading to negative outcomes, including falls, depression and anxiety, cognitive impairment, institutionalization, and mortality [43]. For older adults, chronic health conditions (e.g., cardiovascular disease, diabetes, etc.) and medication use (e.g., benzodiazepines, antidepressants, etc.) contribute to poor sleep quality. These conditions and medications disrupt sleep architecture, causing difficulties in staying asleep and frequent nocturnal awakenings [44]. Managing these issues requires addressing both chronic health factors and medication side effects. Intriguingly, younger adults (18–34 years) had the highest likelihood of experiencing difficulty initiating sleep. Younger adults often experience sleep initiation problems due to lifestyle factors such as excessive screen time, irregular sleep schedules, and late-night engagement with digital devices [45]. The blue light emitted by screens suppresses melatonin production, delaying sleep onset and reducing sleep quality [46]. Increased social media use and academic pressures further exacerbate these issues, leading to poorer sleep hygiene and irregular sleep patterns [47].

Language and cultural background were also significant factors associated with sleep quality and sleep problems. Italian speakers were more likely to report poor sleep quality and difficulty staying asleep than German speakers. Little is known about disparities in sleep problems and quality between different ethnicities [48]. We hypothesize that our findings may reflect social and cultural differences between the two groups, particularly regarding later dinner times and, consequently, later bedtimes among Italian speakers. These behaviors could be associated with lighter or more fragmented sleep. For instance, a European study on meal timing across ten countries found that dinner times were significantly later in Mediterranean countries (typically between 8 and 9 p.m.) compared to Nordic countries (between 4 and 7 p.m.) [49]. Although we are unable to directly test this hypothesis within the scope of the current study, our data show that 14.1% of German-speaking individuals reported going to bed after 11 p.m., compared to 31.1% of Italian-speaking individuals. Further qualitative research is needed to explore the underlying sociocultural mechanisms driving these disparities, including differing attitudes toward sleep health, such as general perceptions of sleep and the importance of maintaining a regular and sufficient sleep routine, to inform tailored sleep health interventions in this region.

Good or very good self-reported health status was consistently associated with better sleep outcomes, whereas chronic diseases increased the likelihood of poor and insufficient sleep. This finding underscores the bidirectional relationship between sleep and health, where poor health may impair sleep and vice versa [50,51]. Sleep medication use was linked to increased odds of all the measured sleep problems, suggesting that, while medications may address acute issues, they are not a sustainable solution for chronic sleep disturbances [52]. Individuals using sleep medications reported poor sleep quality and ongoing sleep issues, consistent with research indicating that such medications provide short-term relief but fail to address the root causes of chronic sleep disturbances. Tolerance, poor sleep hygiene, or a lack of behavioral interventions may contribute to these outcomes. This underscores the need for a comprehensive approach that integrates pharmacological treatments with non-pharmacological strategies such as cognitive–behavioral therapy for insomnia. Public health campaigns should raise awareness about the limitations of sleep medications and advocate evidence-based alternatives to enhance long-term sleep health.

A recent investigation on the use of sedative psychotropic medications from 2019 to 2023 revealed a lower level of benzodiazepine use in South Tyrol than the national Italian average, with a trend toward increased sedative antidepressant use, especially mirtazapine, which likely reflects regional prescription preferences. Z-drug use was similar across both regions, whereas melatonin exhibited a gradual, albeit lower, increase in South Tyrol [53]. Encouragingly, individuals who often or always prioritized getting enough sleep reported significantly fewer sleep problems. This finding emphasizes the importance of sleep hygiene practices and reinforces the potential of behavioral and lifestyle interventions, such as education on sleep health literacy and hygiene, to mitigate sleep disturbances [54].

### 3.1. Implications for Public Health

Our findings underscore the need for and offer valuable insights into potential public health strategies to improve sleep in the South Tyrolean population. Interventions tailored to different cultural groups should address the specific needs of high-risk groups, such as women, older adults, and individuals with chronic illnesses. Public health campaigns that promote sleep health literacy and hygiene may be beneficial [51]. Collaborative campaigns, such as the “Sleep Well, Be Well” from the American Academy of Sleep Medicine, the Centers for Disease Control and Prevention, and the Sleep Research Society, provide interesting examples (https://sleepeducation.org/healthy-sleep/, accessed on 7 May 2025). Within healthcare, general practitioners are well positioned to recognize persistent sleep problems, diagnose sleep disorders, initiate treatments, or refer to sleep specialists [52]. Despite evidence indicating that non-pharmacological treatment is superior in the long-term management of sleep disorders, hypnotics are often considered by general practitioners as the most successful treatment, especially in frail older adults [52]. Therefore, additional efforts to reduce reliance on sleep medications and explore non-pharmacological treatments such as digital cognitive–behavioral therapy for insomnia could improve long-term sleep health in the general adult population.

To reduce sleep medication use, public health interventions should focus on educating both patients and healthcare providers about the benefits of non-pharmacological treatments. Cognitive–behavioral therapy for insomnia (CBT-I) has proven effective in improving sleep and reducing medication use [55]. Digital CBT-I programs, such as Sleepio, offer scalable solutions, and similar initiatives could be implemented in South Tyrol [56]. Additionally, sleep hygiene education, covering lifestyle changes such as screen time management and consistent sleep schedules, can complement CBT-I and promote long-term sleep health [57].

### 3.2. Strengths and Limitations

This study benefited from a large representative sample and the inclusion of diverse demographic and health-related variables. However, this study has some limitations that need to be considered. First, its cross-sectional design limits our ability to establish causality or track changes in sleep quality and problems over time. Future longitudinal studies and objective assessments, such as actigraphy, should provide deeper insights into sleep patterns and their determinants. This study was conducted in South Tyrol, Italy, which may limit the generalizability of our findings to other regions or countries with different healthcare systems. We did not explore potential interactions between independent variables, which could provide further insights into the combined effects of sociodemographic and health-related factors on sleep outcomes. Another limitation of our study was that we did not assess the potential consequences of poor sleep quality, such as sleepiness and alertness, quality of life, impairment at work or school, or impaired interpersonal function.

To effectively utilize longitudinal studies and objective measurement tools like actigraphy and sleep wristbands, research should track participants over time, assessing sleep quality to understand how demographic, lifestyle, and health factors influence sleep. Using actigraphy and sleep wristbands with self-reported data would improve accuracy in measuring sleep duration, efficiency, and nocturnal awakenings. Data should be collected over multiple nights to account for variability and combined with demographic and health data to identify sleep patterns. Additionally, integrating actigraphy or sleep wristband data with other measures, such as heart rate variability, could offer insights into sleep health [58]. Future studies should use data triangulation by combining these objective tools with sleep diaries, questionnaires, and interviews to verify results and enhance reliability.

## 4. Materials and Methods

### 4.1. Study Design

This cross-sectional, population-based survey was conducted jointly by the Provincial Institute of Statistics (ASTAT; Istituto Provinciale di Statistica—Landesinstitut für Statistik, Bolzano-Bozen, Italy) and the Institute of General Practice and Public Health (Bolzano-Bozen, Italy) in the Autonomous Province of Bolzano, South Tyrol.

### 4.2. Setting and Sample

South Tyrol, the Autonomous Province of Bolzano, is part of the Trentino–Alto Adige region in Italy, next to Austria (total population: 534,912), with approximately 70% of the population German-speaking, 25% Italian-speaking, and 5% speaking other languages. The target population of the survey was approximately 400,000 individuals aged 18 years and above residing in South Tyrol. Stratified probabilistic sampling was used in this study. The ASTAT randomly selected adults aged ≥18 years, stratified by age (18–34, 35–54, and 55 and above), gender (male and female), citizenship (Italian or other), and residence (municipalities), from the register of the current resident population in the whole province. To ensure an adequate level of precision, 4000 individuals were sampled considering the distribution of and variation between the strata.

### 4.3. Participant Survey

The participant survey was designed collaboratively by the ASTAT and the Institute of General Practice and Public Health. The German and Italian versions, translated from the ASTAT, were reviewed for language equivalence by a research group at the Institute for General Practice and Public Health.

Sleep quality was measured using the brief version of the Pittsburgh Sleep Quality Index (B-PSQI) [59]. Although polysomnography, actigraphy, or movements captured using other phone apps or body wearables are objective measurements of sleep quality, they are too expensive and impede data collection in large samples. Thus, most epidemiological studies on sleep quality rely on self-reported measures of sleep, such as the Pittsburgh Sleep Quality Index (PSQI) [60]. The original version of the PSQI is a widely used self-report questionnaire designed to measure sleep quality and disturbances over a one-month period [60]. It has been extensively employed in both clinical and research settings and demonstrated reliability and validity across diverse populations and conditions [61,62]. The original version consists of 19 items grouped into 7 component scores. We used the brief version described and tested by Sancho-Domingo et al. (2021) [59], who provided evidence based on internal validity by applying confirmatory factor analyses and sensitivity and specificity for classifying poor sleepers, such as the full PSQI version. The brief version uses six items from the original version to assess five dimensions: perceived sleep quality, sleep duration, sleep efficiency, sleep latency, and sleep disturbances. Thus, the six questions of the B-PSQI yielded five components rated on a scale from 0 to 3, similar to the original version, with higher scores indicating greater sleep disturbance or poorer sleep quality. The component scores were summed to produce a global B-PSQI score ranging from 0 to 15, with scores above 5 indicating poor sleep quality [58]. In our study, we used six items from the original Italian [63] and German versions [20] of the PSQI. Reliability testing revealed acceptable internal consistency for both language versions of the B-PSQI (i.e., Cronbach’s α = 0.74 for the German version; Cronbach’s α = 0.77 for the Italian version).

In accordance with previous population-based studies focusing on sleep issues [64,65], participants’ sociodemographic characteristics included age (birth year), gender (male/female), native tongue (German/Italian/Ladin or others), citizenship (Italy/other country), educational level (below school/high school or higher), community and region of origin (rural/urban), and living situation (alone/with spouse or family member or with parents or children). Health-related factors included self-reported health status (poor, moderate/good, or very good), diagnosed chronic disease (none or nine types of chronic disease), adequate sleep (never, seldom/often, or always), and intake of sleep medications (never/once a week or more often).

### 4.4. Data Collection

Data were collected between 1 March and 30 May 2024. Letters were mailed from the ASTAT to randomly sampled participants to inform them about the study and invite them to voluntarily participate by completing the survey alone or with the aid of a family member. The survey was completed through online self-completion or telephone interviews with collaborators from the ASTAT. One month after the first letter, a second letter was sent to inform the participants about the study and invite them to participate. An online survey was created using LimeSurvey [66].

### 4.5. Statistical Analysis

Descriptive statistics (e.g., frequency) were calculated to describe the measured variables. To address non-response bias and ensure the sample’s representativeness, post-stratification weights were applied to adjust for sociodemographic factors such as age, gender, citizenship, and residence location. Thus, to ensure that the sample closely matched the known distribution of the South Tyrolean population, these post-stratification weights were calculated for each stratum as the ratio of the proportion of the population to the proportion of the corresponding sample using the ISTATsoftware ReGenesees (version 2.3, Rome, Italy). For continuous variables, weighted medians and interquartile ranges were computed; for categorical variables, weighted proportions and 95% confidence intervals were determined. To explore the association between sociodemographic and health-related factors and poor sleep quality, insufficient sleep duration, problems falling asleep, and problems staying asleep, four multivariable binary logistic regression models applying generalized linear modeling were developed. Multicollinearity testing using the variance inflation factor (VIF) revealed no evidence of collinearity among the independent variables (VIF for all variables < 1.4). All analyses were performed using the R Statistical Software (v4.4.2) [67] in the RStudio environment (2024.09.1+394) [68] and the tidyr [69], survey [70], and lme4 [71] packages. A *p*-value of less than 0.05 was considered significant.

## 5. Conclusions

Sleep quality and problems are prevalent among adults in South Tyrol, with significant variations based on gender, age, health status, and sleep behaviors. Public health strategies that promote sleep hygiene and address the needs of vulnerable populations are essential to improve sleep health literacy in the region. Further longitudinal research is warranted to monitor the prevalence of sleep problems and sleep quality at the population level over time and to assess the outcomes and influences of public health campaigns in improving sleep quality and health, as well as the diagnosis and treatment of somnipathies, and reducing the onset and severity of comorbidities associated with disordered sleep.

## Figures and Tables

**Table 1:** Characteristics of the adults participating in the study (N = 2090).

Characteristics	Study Sample (N = 2090)		Total Population (N = 440,134)	
	n	%	n	%
Gender				
Female	1153	55.2	224,019	50.9
Male	937	44.8	216,115	49.1
Age (years)				
18–34	383	18.3	104,337	23.7
35–54	647	31.0	143,802	32.7
55–99	1060	50.7	191,995	43.6
Citizenship				
Italian	2011	96.2	396,997	90.2
Other	79	3.8	43,137	9.8
Community				
Urban	381	18.2	89,445	20.3
Rural	1709	81.8	350,689	79.7
Native tongue				
German	1398	66.9		
Italian	499	23.9		
Ladin or Other	193	9.2		
Living situation ^1^				
Alone	383	18.3		
With partner/family	1326	63.4		
With children	785	37.6		
With parents	196	9.4		
With other family members	124	5.9		
Educational level				
Below high school	1166	55.8		
High school or higher	924	44.2		
Health status				
Poor or moderate	796	38.1		
Good or very good	1294	61.9		
Chronic disease				
None	1282	61.3		
At least one	808	38.7		
Lung diseases (e.g., asthma, chronic obstructive pulmonary disease)	97	4.6		
Cardiovascular diseases (e.g., coronary artery disease, atrial fibrillation, heart failure, stroke/cerebral circulatory disorders)	171	8.2		
High blood pressure	399	19.1		
Kidney diseases	43	2.1		
Immune system disorders	73	3.5		
Cancer	93	4.4		
Metabolic disorders (e.g., diabetes, overweight/obesity)	157	7.5		
Liver diseases	24	1.1		
Mental disorders (e.g., depression, anxiety)	122	5.8		
Taking care to get enough sleep				
Never or seldom	401	19.2		
Often or always	1689	80.8		
Intake of sleep medications during the last month				
Never	1732	82.9		
Once a week or more often	358	17.1		

^1^ Some participants who did not select “living alone” indicated more than one living situation (e.g., living with both a partner and children or living with both parents and other family members). A total of 677 participants (32.4%) reported multiple living situations.

**Table 2:** (a) Differences in sleep duration and sleep quality across demographic groups. (b) Differences in problems falling asleep and staying asleep across demographic groups (N = 2090).

**(a)**				
**Demographic Group (*n*)**	**Sleep Duration (6 h or Less)** ***n* (%) ^2^**	***p*-Value ^3^**	**Poor Sleep Quality ^1^** ***n* (%) ^2^**	***p*-Value ^3^**
Gender		<0.79		<0.001
Female	302 (28.5)		325 (30.6)	
Male	284 (28.0)		212 (20.9)	
Age Group		<0.001		<0.001
18–34 years	106 (21.4)		95 (19.2)	
35–54 years	196 (28.9)		145 (21.5)	
55–99 years	285 (31.4)		296 (32.76)	
Education		0.76		<0.001
Below high school	314 (28.5)		321 (29.2)	
High school or higher	272 (27.9)		215 (22.1)	
Language		<0.001		<0.001
German	327 (24.7)		312 (23.6)	
Italian	189 (39.7)		162 (34.1)	
Ladin or Other	70 (25.5)		62 (22.5)	
Community		0.002		0.04
Urban	146 (34.9)		126 (30.2)	
Rural	440 (26.6)		411 (24.8)	
**(b)**				
**Demographic Group (*n*)**	**Problems falling asleep (>30 min)** ***n* (%) ^2^**	***p*-value ^3^**	**Problems staying asleep (waking up to 3–4 times a week and unable to fall asleep again)** ***n* (%) ^2^**	***p*-value ^3^**
Gender		<0.001		<0.001
Female	117 (11.0)		162 (15.2)	
Male	65 (6.4)		103 (10.0)	
Age Group		0.003		<0.001
18–34 years	58 (11.7)		36 (7.3)	
35–54 years	39 (5.7)		73 (10.7)	
55–99 years	85 (9.3)		155 (17.0)	
Education		0.15		<0.001
Below high school	107 (9.6)		176 (15.9)	
High school or higher	75 (7.7)		88 (8.9)	
Language		0.11		0.03
German	113 (8.5)		156 (11.7)	
Italian	35 (7.3)		79 (16.5)	
Ladin or Other	34 (12.3)		29 (10.4)	
Community		0.52		0.44
Urban	33 (7.8)		59 (13.8)	
Rural	149 (9.0)		206 (12.4)	

^1^ Sleep quality was dichotomized based on the Pittsburgh Sleep Quality Index (B-PSQI) score, with a score above 5 indicating poor sleep quality. ^2^ Weighted percentage with sample-aligned counts based on poststratification weights for age, gender, citizenship, and residence location. ^3^ *p*-values based on the chi-square test.

**Table 3:** (a) Multivariable logistic regression: factors associated with poor sleep quality and insufficient sleep duration (6 h or less) of adults living in South Tyrol (N = 2090). (b) Multivariable logistic regression: factors associated with problems falling asleep (30 min or longer) and problems staying asleep (waking up to 3–4 times a week and unable to fall asleep again) of adults living in South Tyrol (N = 2090).

**(a)**						
**Variables**	**Poor Sleep Quality**			**Insufficient Sleep Duration**		
	**OR**	**95% CI**	***p***	**OR**	**95% CI**	***p***
Females (reference group: males)	2.03	1.63, 2.55	<0.001	1.07	0.86, 1.33	0.6
Age (reference group: 18–34 years)						
35–54 years	1.10	0.78, 1.56	0.6	1.50	1.07, 2.13	0.021
55–99 years	1.69	1.20, 2.39	0.003	1.90	1.35, 2.70	<0.001
Native tongue (reference group: German)						
Italian	1.40	1.05, 1.86	0.023	1.62	1.22, 2.16	<0.001
Ladin and others	1.12	0.75, 1.66	0.6	1.04	0.69, 1.55	0.8
Rural community (reference group: urban community)	1.11	0.81, 1.52	0.5	0.95	0.70, 1.29	0.7
Living alone (reference group: living with family)	1.04	0.79, 1.37	0.8	1.34	1.02, 1.76	0.034
Educational level: high school or higher (reference group: below high school)	0.77	0.60, 0.97	0.027	1.08	0.85, 1.37	0.5
Good or very good health status (reference: poor or moderate)	0.51	0.41, 0.65	<0.001	0.76	0.60, 0.96	0.024
Chronic disease (reference group: none)	1.31	1.03, 1.67	0.027	1.14	0.89, 1.45	0.3
Often or always taking care to get enough sleep (reference group: never or seldom)	0.18	0.14, 0.23	<0.001	0.09	0.07, 0.12	<0.001
Use of sleep medication (reference group: none)	2.27	1.74, 2.95	<0.001	1.37	1.03, 1.81	0.028
**(b)**						
**Variables**	**Problems getting to sleep**			**Problems staying asleep**		
	OR *	**95% CI**	***p***	**OR**	**95% CI**	***p***
Females (reference group: males)	1.91	1.37, 2.69	<0.001	1.96	1.48, 2.61	<0.001
Age (reference group: 18–34 years)						
35–54 years	0.42	0.26, 0.67	<0.001	1.31	0.82, 2.15	0.3
55–99 years	0.60	0.38, 0.94	0.024	1.72	1.09, 2.79	0.023
Native tongue (reference group: German)						
Italian	0.79	0.50, 1.23	0.3	1.30	0.91, 1.84	0.15
Ladin and others	1.82	1.10, 2.95	0.017	1.05	0.61, 1.72	0.9
Rural community (reference group: urban community)	1.50	0.93, 2.52	0.11	1.09	0.75, 1.62	0.7
Living alone (reference group: living with family)	1.05	0.69, 1.55	0.8	0.76	0.53, 1.08	0.14
Educational level: high school or higher (reference group: below high school)	0.77	0.54, 1.09	0.15	0.60	0.44, 0.81	<0.001
Good or very good health status (reference: poor or moderate)	0.50	0.35, 0.70	<0.001	0.54	0.41, 0.72	<0.001
Chronic disease (reference group: none)	1.15	0.80, 1.65	0.4	1.58	1.18, 2.14	0.003
Often or always taking care to get enough sleep (reference group: never or seldom)	0.33	0.24, 0.47	<0.001	0.26	0.20, 0.35	<0.001
Use of sleep medication (reference group: none)	2.03	1.41, 2.90	<0.001	1.67	1.22, 2.27	0.001

* OR: odds ratio, CI: confidence interval.

## Data Availability

Data cannot be shared publicly because of legal restrictions. Anonymized data are available from the Provincial Institute of Statistics (ASTAT) upon request.

## Abbreviations

The following abbreviations are used in this manuscript:

B-PSQI	Brief version of the Pittsburgh Sleep Quality Index
ASTAT	Provincial Institute of Statistics
OR	Odds ratio
CI	Confidence interval
VIF	Variance inflation factor
